# A scalable, dividing cell model for the robust propagation and quantification of human sporadic Creutzfeldt–Jakob disease prions

**DOI:** 10.1073/pnas.2600341123

**Published:** 2026-06-30

**Authors:** Akin Nihat, Parineeta Arora, Christian Schmidt, Melissa L. D. Rayner, Jacqueline Linehan, Sebastian Brandner, Simon Mead, John Collinge, Parmjit S. Jat

**Affiliations:** ^a^ Medical Research Council Prion Unit at University College London, University College London Institute of Prion Diseases, London W1W 7FF, United Kingdom; ^b^ National Prion Clinic, National Hospital for Neurology and Neurosurgery, University College London Hospitals National Health Service Foundation Trust, London WC1N 3BG, United Kingdom; ^c^ Department of Pharmacology, University College London School of Pharmacy, London WC1N 1AX, United Kingdom; ^d^ Division of Neuropathology, the National Hospital for Neurology and Neurosurgery, University College National Health Service Foundation Trust, London WC1N 3BG, United Kingdom; ^e^ Department of Neurodegenerative Disease, University College London Queen Square Institute of Neurology, London WC1N 3BG, United Kingdom

**Keywords:** prion, bioassay, cell culture, Creutzfeldt–Jakob disease, infectivity

## Abstract

Prion diseases represent a unique biological paradigm with mechanistic parallels to other neurodegenerative conditions like Alzheimer’s and Parkinson’s diseases. However, the study of human prion pathobiology and the development of effective therapeutics has been severely constrained by the inability to propagate human prions in dividing cells—forcing reliance on costly and slow animal bioassays. Here, we report the generation of EKV cells—a humanized cell model which supports the robust, indefinite propagation of sporadic Creutzfeldt–Jakob disease (sCJD) prions. We demonstrate that these cells replicate bona fide human prion infectivity in culture—cell lysates induce lethal neurodegeneration in humanized mice that is clinically and neuropathologically indistinguishable from inoculation with sCJD-infected brain tissue. We use EKV cells to develop the Human Prion Assay (HPA), which quantifies sCJD infectivity with sensitivity comparable to gold-standard mouse bioassay, while reducing the experimental timeline from years to weeks. Furthermore, we demonstrate that established sCJD infection can be cured by an anti-prion protein antibody, validating the system as a high-throughput platform for drug discovery. This model bridges a critical translational gap, offering a renewable alternative to animal bioassays, a paradigm to dissect the biology of human sCJD prion disease and screen for therapeutic agents.

## Significance

Developing effective treatments for human prion diseases is severely impeded by the inability to propagate the infectious agent in dividing cell culture. Drug discovery has relied on surrogate tools or protracted animal assays, yielding candidates that fail in clinical trials. Here, we describe EKV cells, a scalable dividing cell line that supports the robust propagation of infectious sporadic Creutzfeldt–Jakob disease (sCJD) prions. These cells maintain the core strain-specific signature of the human pathogen and enable direct infectivity quantification with sensitivity comparable to gold-standard mouse bioassays. By establishing a platform that validates the clearance of authentic human prion infection, this model bridges a critical translational gap, offering a powerful tool to dissect human prion biology and identify therapeutics with direct clinical relevance.

Prion diseases are a group of uniformly fatal, progressive neurodegenerative conditions, of which the most common human form is sporadic Creutzfeldt–Jakob disease (sCJD) (1). Prion diseases are caused by the autocatalytic, self-templated misfolding of the constitutive cellular prion protein (PrP^C^) into prions—assemblies of disease-associated isoforms, some of which acquire protease resistance (PrP^Sc^) (2).

Prions exist as “clouds” of conformational variants, or quasispecies, which can be preferentially selected for under different environmental, experimental, or genetic pressures (2, 3). Distinct PrP conformations give rise to disease strains (4) that cause conserved clinicopathological profiles, a phenomenon increasingly recognized in other neurodegenerative proteinopathies—including the identification of distinct proteopathic conformers in phenotypically diverse tauopathies like Alzheimer’s disease and Progressive Supranuclear Palsy (PSP), and synuclein conformers in Parkinson’s disease and Multiple System Atrophy (MSA) (5). A number of strains are recognized in sCJD, defined in part by the polymorphism at codon 129 (methionine/valine) of the prion protein gene (*PRNP*) (6).

The study of human prion biology has been severely constrained by the inability to transmit and propagate the infectious agent in cell culture, despite several decades of determined effort (7). Moreover, the reliance on screening platforms that do not recapitulate the biological nuances of human prion propagation is likely to underlie the failure in humans of many putative anti-prion agents (8)—noting that the mechanisms by which prions enter, spread, and are removed from cells are strain-specific (9). Consequently, the field has relied on surrogate tools: these include transgenic mouse models, which faithfully replicate human disease (10) but are prohibitively expensive, require specialized biological containment level 3 animal husbandry facilities and require up to 2 y to deliver results; or seed amplification assays (SAAs) like Real-Time Quaking-Induced Conversion (RT-QuIC). RT-QuIC offers rapid diagnostic accuracy in sCJD but does not reflect biological disease activity or infectivity (11). The dissociation between seeding activity and true infectivity remains a significant barrier to identifying therapeutics that might arrest the replication of the infectious human prions responsible for disease (12). Cell-based assays for murine prions, such as the Scrapie Cell Assay (SCA) (13), revolutionized the study of nonhuman prions by permitting high-throughput quantification and correlation with biological activity (14, 15). However, the lack of an equivalent system for propagating human prions has precluded similar advances in understanding their biology.

To address this translational gap, we aimed to engineer a dividing cell line capable of sustainably replicating infectious human sCJD prions. We hypothesized that expressing human PrP in a murine cell line with broad nonhuman prion strain tropism would provide an environment supportive of sCJD replication, which could be further enriched for as-yet unknown prion susceptibility factors by iterative single cell cloning.

Here, we report the development of the EKV cell line, a humanized murine cell model that propagates bona fide infectious sCJD prions in dividing cells. We demonstrate that EKV cells reproducibly produce high-titer infectious sCJD prions that cause lethal prion disease in humanized mice indistinguishable from inoculation with human sCJD-infected brain tissue. By using these cells to establish a cell-based Human Prion Assay (HPA) that can quantify human sCJD prion infectivity and demonstrate clearance of established infection with an anti-PrP antibody, we develop a scalable platform to directly investigate human prion biology, identify novel therapeutic compounds and provide a renewable source of sCJD prions that recapitulate key pathological features of brain-derived prions.

## Results

### Developing the EKV Cell Line, a Dividing Cell Model of sCJD.

To generate a dividing cell system to propagate human prions, we utilized CAD5 cells—a murine central nervous system catecholaminergic cell line previously shown to support the replication of diverse murine prion strains (16). CAD5 prion tropism was recently extended to other animal strains by expressing homologous PrP in place of the endogenous mouse prion protein (17). To prevent dominant-negative inhibition of human prion propagation (18), we stably silenced endogenous mouse PrP expression in CAD5 cells using RNA interference (19), deriving a CAD5 PrP-knockdown cell line (CAD5-KDB3, KDB3). KDB3 cells express PrP RNA at <1% level of CAD5 wild-type cells, and when reconstituted with the full-length open reading frame of mouse PrP, are susceptible to mouse-adapted 22L, RML, ME7, and MRC2 prion strains respectively (19).

Transgenic mice expressing human *PRNP* with valine at codon 129 (V129), instead of the murine form, are susceptible to all human prion strains, regardless of the *PRNP* codon 129 polymorphism (20). We therefore hypothesized that expressing V129 human PrP in CAD5 cells would provide a favorable replication environment for sCJD prions (21), which could subsequently be enriched without prior knowledge of the necessary susceptibility factors. We reconstituted CAD5-KDB3 cells with the full-length human *PRNP* open reading frame encoding valine at codon 129. To optimize prion protein trafficking and expression in murine cells, the human signal peptide was replaced with the mouse equivalent (HuPrPmssV129). After retroviral transduction, we undertook an exhaustive single cell clone screening process, isolating 480 clones to screen for susceptibility to infection with sCJD prions. Given the absence of defined cellular factors governing cellular replication of human prions (8), we reasoned that susceptibility is likely determined by rare, stochastic combinations of cellular cofactors that support one or more of the cloud of prion conformations contained within a tissue sample—screening a bulk cell population would therefore mask highly permissive but rare cell clones.

**Fig. 1.**
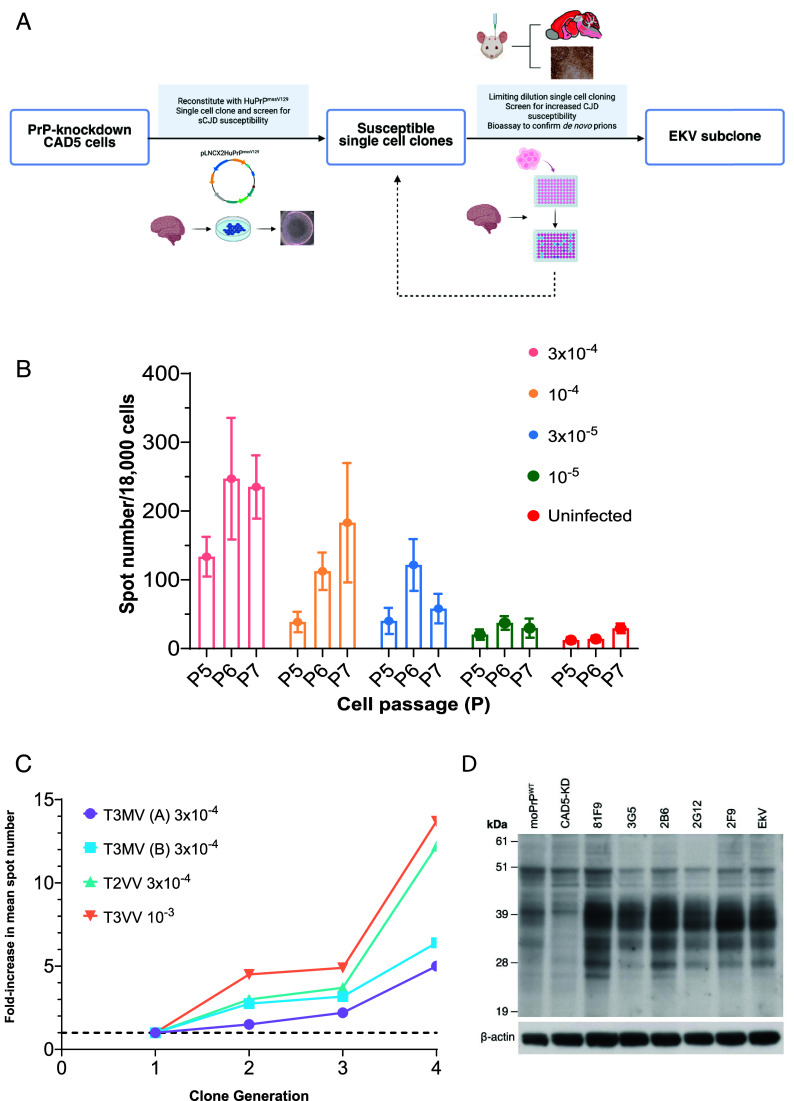
Iterative development of CAD5 HuPrPmssV129 clones susceptible to sporadic CJD prions. (*A*) Schematic representation of the engineering, cloning, and screening of CAD5 HuPrPmssV129 clones and development of the EKV cell line. CAD5-KDB3 cells, with mouse PrP expression knocked down, were reconstituted with the entire open reading frame of human *PRNP* (valine at codon 129) and the mouse signal peptide. Single cell clones were prepared and challenged with sporadic Creutzfeldt–Jakob (sCJD) T3MV/T2MM prions in an SCA format to identify susceptible clones. The SCA was repeated in a multiwell format to ensure the clones were reproducibly susceptible. One such clone, 81F9, was taken forward and iteratively single cell cloned and screened for increasing sCJD prion susceptibility, leading to EKV, the optimal clone. In parallel, sCJD-exposed 81F9 cells were subjected to Tg152c mouse bioassay to confirm propagation of de novo infectious prions. (*B*) Susceptibility of the selected first-generation single cell clone, 81F9, to T3MV sCJD. Twelve replicate wells in 96-well format were challenged with four dilutions of sCJD T3MV frontal cortex homogenate (I4960, weight/volume dilutions), passaged seven times to clear the initial inoculum, and subjected to PK-digestion and ELISPOT revelation with ICSM18 antibody. Cell susceptibility was measured as PrP^Sc^ spot number per well and is displayed as mean spot number and SD. (*C*) Relative increase in susceptibility to infection with sCJD prions of the best clone from each generation of single cell clones. Four cell clones were challenged with T2/T3 sCJD prion-infected frontal cortex homogenate (valine hetero- or homozygous at codon 129) at the dilutions shown, passaged four times and subjected to ELISPOT assay. Mean spot count of 8 replicate wells per clone was normalized to fold-change from the 1st generation clone, 81F9 (dotted line). (*D*) Representative immunoblots of PrP^C^ expression in engineered CAD5 lines and subclones: CAD5 PrP-knockdown cells (CAD5-KD, lane 2), reconstituted with full-length MoPrP (moPrP^WT^, lane 1), the first selected HuPrPmssV129 clone (81F9, lane 3) and selected single cell clones with increased susceptibility to sCJD (3G5, 2nd generation; 2B6/2G12, 3rd generation; 2F9/EKV, 4th generation). 15 µg protein in 15 µL was loaded per lane, blots were developed with anti-PrP antibody ICSM35 and stripped/reprobed with mouse anti-β actin antibody as a loading control.

The single cell clones were challenged with two brain homogenates from patients with sCJD, initially identifying a small subset of clones that appeared to accumulate disease-associated PrP^Sc^ over limited cell passage. We selected the most promising candidate, 81F9, for further optimization. This clone reproducibly propagated modest amounts of PrP^Sc^ after challenge with a brain homogenate containing type 3 strain sCJD prions (codon 129 methionine/valine heterozygous, T3MV London classification (6)—equivalent to types 1 and 2 in the widely used international classification system (22), Fig. 1*B*). However, exposure to multiple homogenates containing type 2 [codon 129 methionine homozygous, T2MM (6)] yielded heterogenous and poorly reproducible results. We hypothesized that the *PRNP* codon 129 genotype mismatch between the inoculating prion strains (methionine homozygous, MM) and host (valine homozygous, VV) resulted in a prion replication environment too inefficient to consistently overcome normal cellular prion removal and division. We therefore focused further cell clone development on brain homogenates from patients with at least one *PRNP* codon 129 valine allele.

To isolate a highly prion-susceptible subclone, we undertook iterative limiting dilution single cell cloning coupled with screening for sCJD propagation at increasing dilutions of infected brain homogenate, effectively directing 81F9 evolution toward increased sCJD susceptibility. This process yielded the fourth-generation EKV subclone (Fig. 1*A*), which accumulated 5 to 14 times more PrP^Sc^ than the parental 81F9 cells, after challenge with multiple sCJD brain homogenates (Fig. 1*C*). Immunoblotting of sequential single cell clones demonstrated that EKV cells expressed PrP at comparable levels to less susceptible clones, suggesting that the increased prion susceptibility was not simply a function of increasing PrP expression (Fig. 1*D*); this implies the enrichment of unidentified cellular factors favorable for human prion replication.

### Propagation of De Novo, Infectious Human sCJD Prions.

Infectivity is a defining feature of prions (23); in cell-based assays this is classically measured via the presence of proteinase K (PK)-resistant PrP^Sc^ aggregates, which should first be correlated with genuine prion biological activity (13). A central challenge in developing cell models of any neurodegenerative proteinopathy is demonstrating that a cell model is propagating a biologically relevant (in this case, infectious) agent that causes disease (5), rather than accumulating inert or inconsequential protein aggregates that might develop spontaneously or following gene overexpression or other cell manipulation. To definitively prove that HuPrPmssV129 cells were propagating bona fide infectious prions, we undertook a gold-standard transmission bioassay in transgenic mice.

**Fig. 2.**
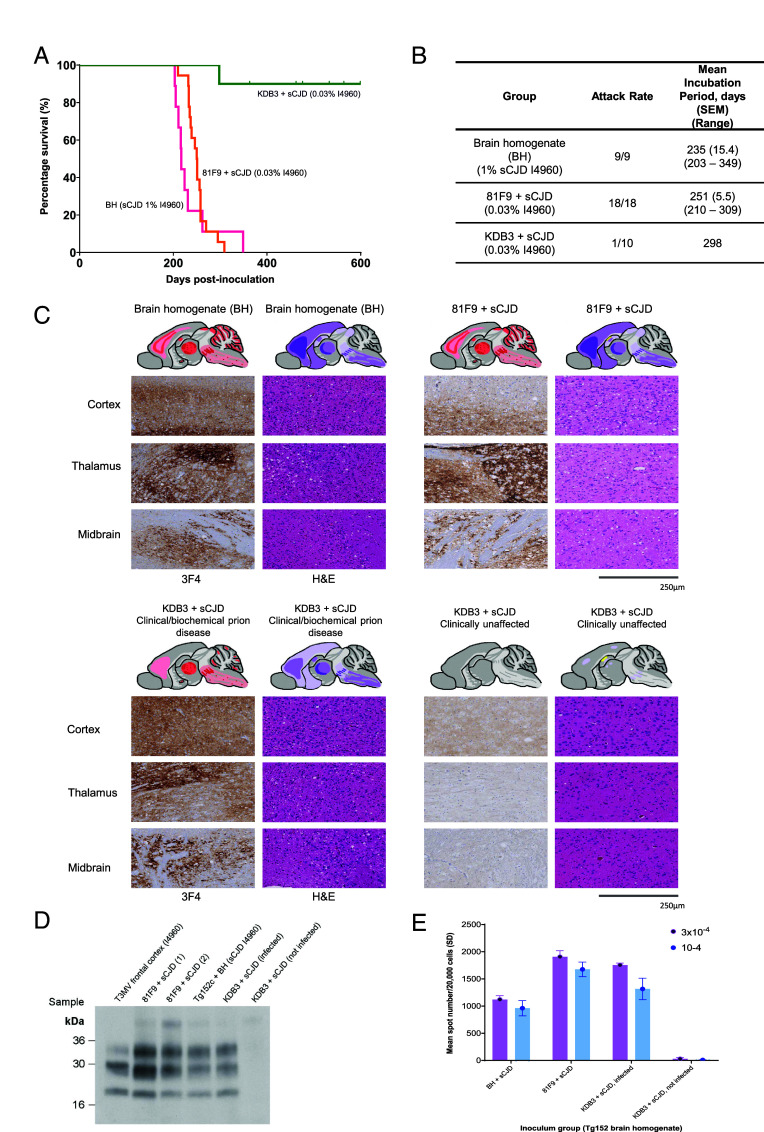
Engineered CAD5 cells propagate de novo, infectious human sCJD prions, with preserved biochemical properties. (*A*) Kaplan–Meier survival curves for Tg152c mice inoculated with 1% brain homogenate from T3MV sCJD sample I4960 (BH), pooled 81F9 cell lysate after challenge with 0.03% I4960 homogenate and serial passage (81F9 + sCJD), or CAD5 PrP-knockdown KDB3 cell clone challenged with 0.03% I4960 homogenate (KDB3 + sCJD) and serially passaged. Both BH and 81F9 + sCJD curves were significantly different from KDB3 + sCJD (*P* < 0.0001, log-rank with Bonferroni correction), but not from each other (*P* = 0.30). (*B*) Summary of clinical and histopathological attack rates, and mean incubation period by group. (*C*) Neuropathological analysis of Tg152c (HuPrP V129) transgenic mouse brain after inoculation with sCJD-infected frontal cortex homogenate or sCJD-challenged cell homogenate. Each inoculum group is represented by three paired images of immunohistochemistry (anti-PrP antibody 3F4, left columns) demonstrating PrP deposition, and H&E stain (right columns) for neuronal loss and spongiosis, from cortical, thalamic, and midbrain areas. Schematic drawings show the regional distribution of PrP deposition (diffuse synaptic staining in light red, the darker shade indicates greater deposition; microplaques as dark red spots) and spongiosis (purple; mild-severe according to darker shade). Scale bar, 250 µm; hematoxylin & eosin, H&E. (*D*) Representative immunoblots of Tg152c brain homogenate from each group (81F9 + sCJD group represented with two samples, (1 and 2), from separate mice. I4960 T3MV sCJD frontal cortex shown in lane 1 as a control. (*E*) Inoculation of Tg152c mouse brain homogenate into prion-naïve, highly sensitive EKV cells to assess for occult infectivity; KDB3 + sCJD (infected), brain homogenate from the single mouse with symptomatic and histopathological prion disease from the KDB3 + sCJD group; KDB3 + sCJD (not infected), representative brain homogenate from KDB3 + sCJD mice (n = 9), without histopathological or clinical prion disease. Displayed are means/SD of 6-well replicates from two independent experiments, brain homogenate challenge at two dilutions (3 × 10^−4^ and 10^−4^).

We inoculated humanized Tg152-congenic (Tg152c) mice [which overexpress human V129 PrP on a mouse *Prnp*-null background (24)] with cell lysates from first-generation 81F9 and CAD5-KDB3 cells, after challenge with sCJD brain homogenate and six cell passages to dilute out the original inoculum. Results clearly demonstrated that 81F9 cells propagated genuine, infectious sCJD prions (Fig. 2 *A* and *B*): 100% (18/18) of cell lysate-inoculated mice succumbed to prion disease, with a mean incubation period (251 ± 6 d postinoculation, DPI) similar to mice inoculated with the original sCJD-infected brain homogenate (9/9 clinically affected mice, mean incubation 235 ± 15 DPI). Most importantly, the neuropathological phenotype of the cell-derived prion disease was indistinguishable from that of the human brain homogenate-inoculated group (Fig. 2*C*), characterized by widespread moderate spongiosis (most pronounced in the thalamus and brainstem) and diffuse PrP^Sc^ synaptic deposition, with microplaques predominantly in the thalamus and midbrain. Biochemical strain typing also confirmed that the cell-propagated prions retain the strain-specific properties of the original sCJD brain homogenate inoculated directly into transgenic mice (Fig. 2*D*). Together, these results demonstrate that the 81F9 cell clone propagates bona fide infectious sCJD prions in cell culture.

Unexpectedly, one mouse from the group inoculated with lysate from PrP-knockdown (KDB3) cells exposed to sCJD (1/10 mice, incubation period 298 DPI) also developed clinical and neuropathological prion disease (Fig. 2 *A*–*C*), confirmed on immunoblotting (Fig. 2*D*); the other nine mice in this group did not. Four mice from this group died during the experimental period of other causes (363 to 533 DPI), and none showed biochemical or histopathological evidence of prion disease. We considered that clinically unaffected mice might harbor occult prion infectivity, and tested this by exposing highly prion-susceptible EKV cells to brain homogenate from clinically affected and unaffected mice from the KDB3 lysate-inoculated group (Fig. 2*E*). As expected, the clinically and biochemically affected mouse brain homogenate caused stable accumulation of PrP^Sc^ in EKV cells, while homogenate from unaffected mice did not. Tg152c mice do not spontaneously generate prions, suggesting that the sCJD-exposed KDB3 cells harbored a very low level of residual prion infectivity from the original brain homogenate even after six cell passages.

### The HPA: A Rapid, Scalable Method to Detect and Study Human Prions.

Mouse bioassays require large cohorts of animals, specialized and expensive biosafety facilities, and typically take 1 to 2 y to deliver definitive results—considerably limiting the investigation of many basic aspects of human prion biology. Having established that 81F9 cells propagate authentic, infectious sCJD prions which could be detected via ELISPOT assay, we therefore next sought to use the more susceptible, 4th generation EKV clone to develop a quantitative Human Prion Assay (HPA) that could act as a robust alternative to the gold standard transgenic mouse bioassay.

**Fig. 3.**
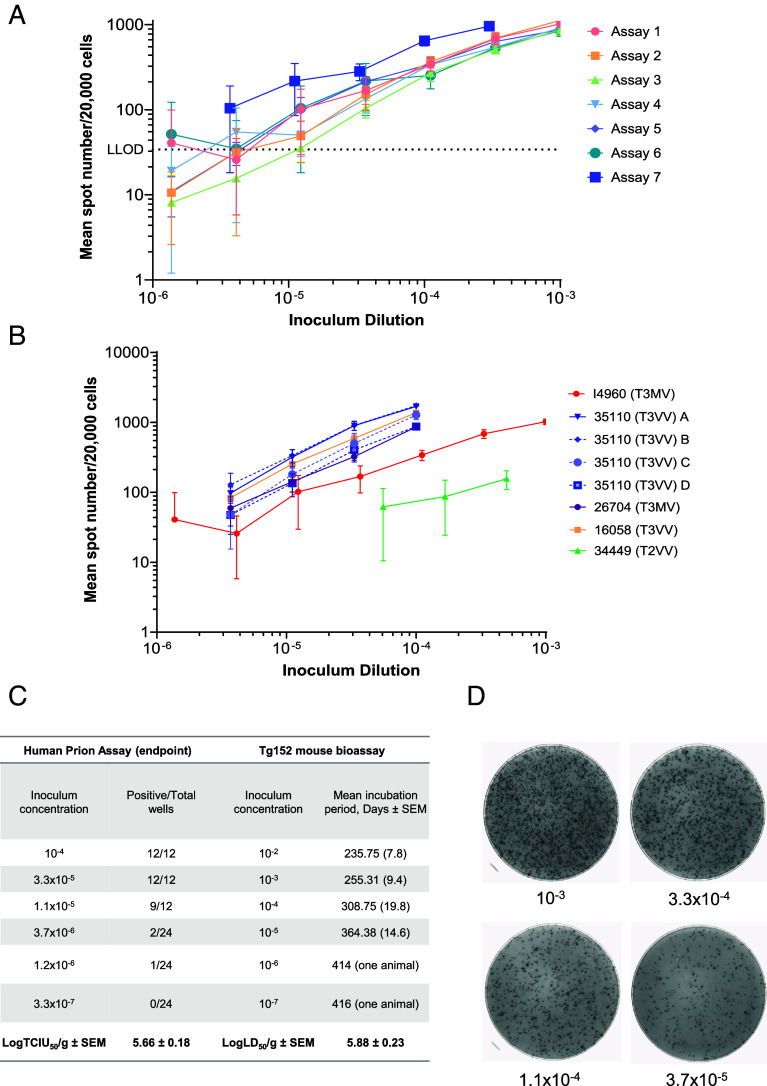
Establishment of a cell-based HPA to measure sCJD infectivity from the human brain. (*A*) Linear and dynamic range of the HPA over serial assays. Seven independent assays were undertaken over 9 mo, using separate aliquots of the same sCJD frontal cortex homogenate (T3MV, I4960). EKV cells were challenged with serial 1:3 dilutions of I4960 (12 wells per dilution, 10^−3^ to 1.37 × 10^−6^), passaged 4 times and infectivity quantified (mean PrP^Sc^ spot number and SD per 20,000 cells). HPA lower limit of detection (*LLOD, 34 spots per well*) was calculated as the pooled mean of uninfected wells (12 wells per assay, 20,000 cells per well filtered to ELISPOT) + 3× pooled SD. HPA linear range (3.3 × 10^−4^ to 1.1 × 10^−5^) was defined as the inoculum dilution range resulting in a strong linear relationship (R^2^ > 0.9, mean across seven assays 0.94 ± 0.02) between PrP^Sc^ spot number and dilution above LLOD, across all assays on a double logarithmic plot. (*B*) HPAs measuring infectivity from five different sCJD frontal cortex homogenates (T2/3, MV/VV). EKV cells (12 wells per dilution) were challenged with serial dilutions of five sCJD frontal cortex homogenates: four independent assays with different homogenate aliquots of 35110 (T3VV); I4960 (Assay 1 from Fig. 1*A*); 26704 (T3MV); 16058 (T3VV); 34449 (T2VV). Cells were passaged 4 times and PrP^Sc^ quantified as outlined previously. Dotted lines represent different aliquots of I4960 and 35110, assayed in independent experiments. (*C*) Comparative sensitivity and estimated sCJD infectious titer by endpoint HPA and gold-standard transgenic mouse bioassay. In the endpoint HPA, EKV cells were challenged with 300 µL serial 1:3 dilutions of I4960 (T3MV) sCJD frontal cortex homogenate (12 to 24 wells per dilution), passaged seven times, and PrP^Sc^ quantified; wells were recorded as positive if the spot number was greater than the mean spot number of uninfected EKV cells + 10 × SD (12 wells per assay). Displayed are the number of positive and total wells per dilution from one of three independent experiments. For the mouse bioassay, Tg152c mice expressing human PrP (valine at codon 129) were inoculated intracerebrally with 30 µL serial 1:10 dilutions of I4960 brain homogenate (three groups of 20 mice per dilution, each group inoculated with a different aliquot). The mean incubation period per group is shown for serial inoculations of one of three I4960 aliquots into groups of 20 mice. Infectious titers were calculated using a modified Karber method (25), expressed as log tissue culture infectious units (LogTCID_50_/g brain homogenate) for the HPA (mean of three independent experiments ± SEM), and log lethal dose (LD_50_/g brain homogenate) for the mouse bioassay (mean of three inoculation groups, ± SEM). (*D*) representative ELISPOT wells showing PrP^Sc^-positive spots for EKV cells challenged with serial dilutions of I4960 brain homogenate.

We therefore adapted EKV cells to a 96-well HPA, analogous to the widely used Scrapie Cell Assay (SCA) for nonhuman prions (13). Using multiple replicate assays, we rigorously established robust detection of infectious sCJD prions over two orders of magnitude of inoculum concentration (10^−3^ to 10^−5^), with excellent signal to noise ratio allowing detection of sCJD prions from the sampled crude brain homogenate when diluted 100,000-fold (mean assay lower limit of detection 1.02 × 10^−5^ dilution, Fig. 3*A*). We next demonstrated that the HPA could detect and propagate prions from a range of sCJD crude brain homogenates infected with both type 2 and 3 prion strains (from patients carrying at least one valine allele at codon 129), with relative titer varying reproducibly by up to two orders of magnitude across homogenates (assay R^2^ > 0.97, Fig. 3*B*). Although EKV cells were more susceptible to prions from all assayed brain homogenate samples compared to the parental 81F9 clone, they did not accumulate PrP^Sc^ after challenge with brain homogenate containing type 2 sCJD from patients homozygous for methionine at codon 129—supporting our hypothesis that a host and inoculum *PRNP* codon 129 genotype mismatch reduces sCJD replication efficiency in these cells.

To validate the HPA against the in vivo gold standard mouse bioassay, we performed a side-by-side endpoint titration of a single sCJD isolate. The infectious titer calculated by the EKV HPA (5.66 ± 0.18 logTCIU_50_/g) was comparable to the mouse bioassay-derived titer (5.88 ± 0.23 logLD_50_/g, Fig. 3*C*)—however, whereas the mouse bioassay required a significant number of animals and took approximately 500 d to complete, the cell-based bioassay was over tenfold faster, avoided animal use, and was conducted at a fraction of the cost.

### Establishing a Scalable Platform for Therapeutic Screening.

**Fig. 4.**
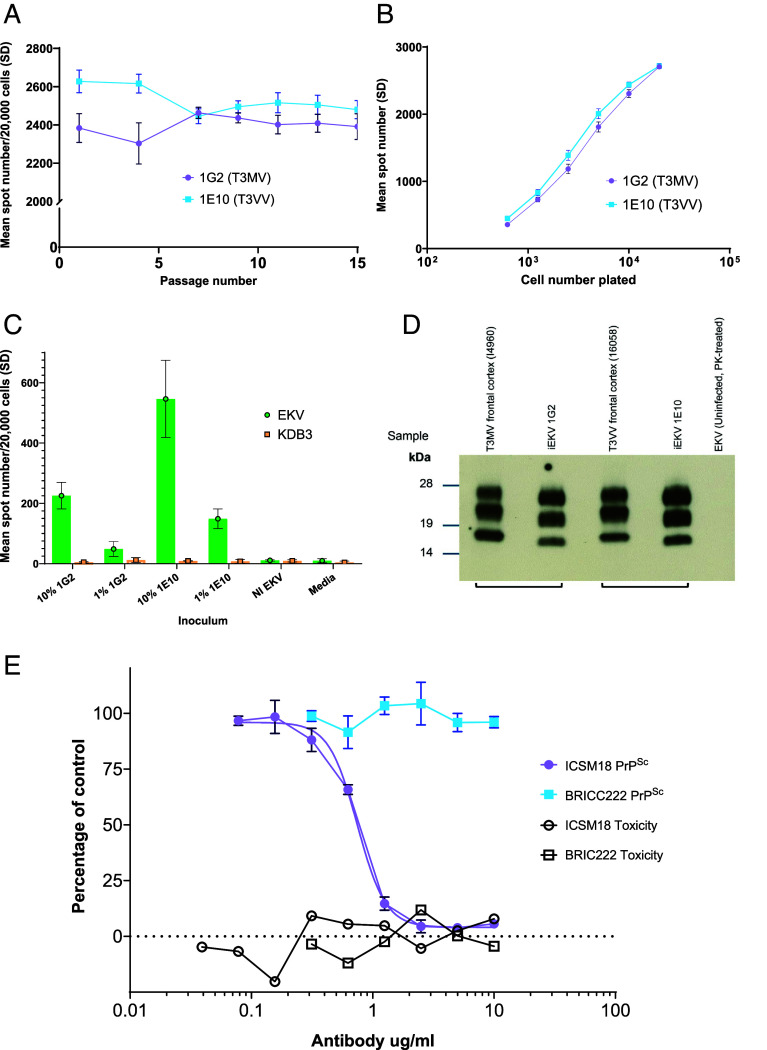
EKV cells can persistently propagate infectious human prions, and demonstrate efficacy of an anti-prion agent. (*A*) Long-term propagation of human prions in EKV cells. Two selected iEKV subclones infected with T3MV (1G2) and T3VV (1E10) prion samples were serially passaged in 6-well replicates, and PrP^Sc^ assayed on alternate passages (20,000 cells). (*B*) PrP^Sc^ spot count in 1G2 and 1E10 clones is a function of plated cell number. PrP^Sc^ load was quantified in serial 1:2 dilutions of 1G2 and 1E10 clones in 6-well replicates; mean PrP^Sc^ spot count (representing a single infected cell) for 1G2 was 50% ± 10% total cell count, and 59% ± 14% for clone 1E10. (*C*) Cell homogenate from iEKV clones can transmit infectivity to naïve EKV cells, but not PrP-knockdown KDB3 cells. 10^7^ cells of each clone were resuspended in 1 mL D-PBS and ribolyzed. Uninfected EKV cells were challenged with 10% and 1% cell homogenates in media passaged four times and PrP^Sc^ quantified, as described. NI EKV, Noninfected EKV cells were controls. (*D*) Immunoblot of persistently infected iEKV cell subclones 1G2 (sCJD T3MV, homogenate I4960) and 1E10 (sCJD T3VV, homogenate 16058) alongside the original inoculating homogenates and PK-treated uninfected EKV cells. Unlabeled wells are blank. Solid bars below the immunoblot indicate paired samples (the respective iEKV subclone with its original inoculum). (*E*) Treatment of 1G2 iEKV cells with anti-prion monoclonal antibody ICSM18. At confluence, 10,000 1G2 cells were seeded in 6 replicates to wells of a 96-well microplate, allowed to settle for 2 h, then incubated with a dilution series of ICSM18 or its isotype control, BRIC222 antibody. After 5 d, media was gently replaced and 7,000 cells per well filtered for PrP^Sc^ ELISPOT detection, or cell viability assay. Untreated iEKV cells, noninfected EKV cells, media-only wells, and DMSO-treated iEKV cells were used as experimental controls. PrP^Sc^ and toxicity were normalized as a percentage of untreated iEKV cells in 4 to 6-well replicates. Displayed are representative results from one of three independent experiments.

A critical bottleneck in human prion disease drug discovery is the lack of a screening cell model to predict therapeutic efficacy. To establish the EKV cells as a therapeutic screening platform, we generated persistently infected cell lines (iEKV) by subcloning cells after exposure to two separate sCJD-infected brain homogenates. The resultant clones maintained stable propagation of sCJD prions (T3MV iEKV clone 1G2, mean infected cells 50% ± 10%; T3VV iEKV clone 1E10, mean infected cells 59% ± 14%) over at least 15 cell passages (40 to 55 d, Fig. 4 *A* and *B*). iEKV cells can be cryopreserved and resuscitated with minimal loss of prion infectivity. Moreover, lysates from iEKV cells efficiently reinfect prion-naïve EKV cells, but not PrP-knockdown KDB3 cells (Fig. 4*C*), and produce PK-resistant PrP^Sc^ with comparable glycoform patterns to the original infecting sCJD inoculum (Fig. 4*D*)—establishing a renewable, cell-derived source of infectious sCJD prions that broadly preserve the biochemical characteristics of the inoculating prions, without the need for additional human or animal tissue.

To demonstrate the utility of this platform for drug screening using a known prion inhibitor, we undertook a pilot treatment trial using the anti-PrP monoclonal antibody ICSM18, which has previously shown efficacy against nonhuman prions in culture (26) and in vivo (27). ICSM18 treatment resulted in a potent, dose-dependent clearance of human PrP^Sc^ in iEKV cells (Fig. 4*E*), with an EC_50_ of 4.7 nM ± 0.40 (over three independent assays), which is approximately 6-fold higher than previously reported in murine prion-infected cells (26). An isotype control antibody had no effect on PrP^Sc^ levels. Unlike previous therapeutic screens, which have relied on murine prions and identified compounds like quinacrine that subsequently failed in human trials (28, 29), iEKV cells provide a means for high-throughput screening of small molecule inhibitors directly against genuine infectious human prions.

## Discussion

The establishment of the EKV cell line addresses a fundamental barrier to understanding human prion pathobiology and developing effective therapeutics—the inability to replicate, measure, and study genuine infectious human prions in culture (8). We show that humanized, dividing murine cells can be robustly infected with and propagate bona fide, de novo sCJD prions that retain strain characteristics and are indistinguishable from the original inoculum upon passage into gold-standard transgenic mouse bioassays. By demonstrating that EKV cell-produced prions recapitulate the core biological features of human disease, we establish a scalable platform to directly quantify human sCJD prions and undertake high-throughput therapeutic screens, circumventing the current reliance on animal bioassays and bridging a translational gap that has persisted for decades (7).

A defining requirement for any relevant model of neurodegenerative proteinopathies is demonstrating that it propagates biologically active species rather than inert or clinically inconsequential aggregates. We definitively show that EKV cells produce genuine human prions with quantifiable infectivity that can be differentiated from the original sCJD-infected inoculum after serial cell passage. This distinguishes EKV cells from recent nondividing cell models, such as human induced pluripotent stem cell (iPSC)-derived astrocytes (30) or cerebral organoids (31). While these models offer valuable access to human cellular microenvironments, their utility is constrained by their heterogeneity, technical complexity, and low throughput (32). Crucially, because cells cannot be serially passaged to dilute the original inoculum, it is impossible to confirm genuine prion production, quantify titer, or distinguish products from the inoculating prion species, which can persist even in the absence of PrP expression (33).

Using EKV cells, we developed the HPA, which offers sensitivity comparable to the gold-standard transgenic mouse bioassay but provides results approximately ten times faster and at a fraction of the cost. Importantly, the HPA supports the replacement and reduction of animal use in human prion research, by offering a credible alternative for determining infectious sCJD prion titer. While the current HPA detection limit (approximately 10^−5^ dilution) may not yet support diagnostic use for tissues or biofluids with low prion titers (34), this system will allow high-throughput quantification of human prion infectivity in brain tissue, opening routes to addressing research questions that were previously intractable due to logistical constraints. Several established methods to concentrate prion infectivity or enhance cellular uptake provide promising strategies to further increase detection sensitivity (35, 36).

The failure of putative anti-prion agents in human trials, despite efficacy in murine cell-based assays, highlights the need for human prion-specific therapeutic screening tools. We demonstrate that chronically infected iEKV cells maintain stable sCJD infection over extended passage and can be cured by the anti-PrP monoclonal antibody ICSM18, validating the system as a platform to identify and investigate compounds that specifically target human prion replication in high-throughput. This measures the neutralization of biological infectivity directly, allowing us to rigorously distinguish between drugs that suppress amyloid formation—which might be identified by screening systems using SAAs like RT-QuIC to detect amyloid (37)—and those that disrupt genuine prion infectivity.

The specific requirements for productive cellular prion infection remain elusive. While PrP expression is essential, it is neither sufficient nor correlated with susceptibility (38). Indeed, we observed no consistent increase in human PrP expression in more susceptible subclones—although a formal quantitative comparison has not yet been undertaken. It would be useful in future to confirm these findings, and also establish if EKV cells require higher levels of human PrP expression for prion susceptibility than endogenous mouse PrP in wild-type CAD5 cells. In immortalized cells, where prion transmission is primarily vertical (39), it is axiomatic that stable propagation requires a replication rate that outstrips removal via cell division and active recycling pathways (21, 40). Contrary to the hypothesis that human prions simply propagate too slowly for dividing cells (41), we found no difference in doubling time between more or less-permissive subclones—arguing that this is not a major driver of increased susceptibility.

Previous studies have implicated PrP^C^ cell surface availability (42) and extracellular matrix composition (43), yet no unifying factors have emerged. Cell permissiveness and turnover in nonhuman prions is demonstrably strain-specific (44, 45), and EKV cells offer the opportunity to empirically determine if human sCJD prion infection relies on similar diverse mechanisms of cellular uptake and trafficking, and what the determinants of susceptibility are. Interestingly, similar attempts to engineer human prion-propagating cells in other prion-susceptible cells have failed (46), suggesting that unique host factors might underlie the broad prion strain tropism of CAD5 cells. They exhibit a distinct PrP interactome (47), marked by lower levels of basal autophagy and increased exosome release (48). Importantly, our iterative subcloning strategy enriched for sCJD prion susceptibility across all assayed homogenates, rather than selecting for specific isolates. This implies the enrichment of broadly relevant susceptibility factors, which might be determined via comparative genomic and transcriptomic analysis of sequential subclones.

There are a number of limitations to this work. We can demonstrably detect PK-resistant PrP by immunoblot in iEKV cells, which have been subcloned after sCJD challenge to select for persistent prion propagation. However, we have not directly undertaken immunoblot of naïve EKV cells challenged with sCJD and passaged as part of the HPA—as they typically have a lower percentage of infected cells than iEKV subclones, it is possible that the total PrP^Sc^ amount may be below the detection threshold for less sensitive biochemical assays like Western blotting. PK-resistant PrP from sCJD-infected iEKV cells also demonstrates a slight downward shift in band migration compared to the original inoculum (Fig. 4*D*), similar to T3MV sCJD prions passaged through Tg152c mice (Fig. 2*D*), suggesting a degree of host adaptation. Future work should aim to directly identify and further characterize the biochemical properties of PK-resistant PrP in newly-infected EKV cells, using extended cell passage and biochemical methods to increase sensitivity of detection if required (49).

EKV cells were derived from the PrP-knockdown KDB3 line, which continues to express <1% levels of wild-type CAD5 PrP RNA. Coexpression of PrP orthologs can reduce prion replication efficiency via dominant-negative inhibition (18), which may have reduced the likelihood of detecting sCJD-susceptible subclones. We have since employed a complementary *PRNP*-knockout strategy utilizing CRISPR/Cas9 technology to develop dividing cells that are susceptible to variant CJD (50). Finally, we identified during transgenic mouse bioassay that six cell passages may be inadequate to completely remove all residual sCJD inoculum from culture. This is below the detection limit of the HPA (as demonstrated by the absence of increased ELISPOT signal on sCJD-exposed KDB3 cells) and will therefore not affect assay results; however, it underlines the importance of extended serial cell passage and inclusion of appropriate assay controls to accurately quantify prion infectivity.

EKV cells expressing human PrP (V129) propagated sCJD prions solely from inocula possessing at least one valine allele at *PRNP* codon 129, but do not propagate sCJD prions from methionine homozygous (MM) patients. This recapitulates the relative transmission barrier observed in HuPrP(V129) transgenic mice, where MM sCJD transmission is characterized by prolonged incubation periods (51). While this might suggest a kinetic barrier, CAD5 subclones expressing human PrP(M129) that propagate highly infectious MM variant CJD prions are resistant to MM sCJD prions (50). Consequently, the failure of CAD5-derived lines to replicate MM sCJD is likely not driven by genotype mismatch or replication kinetics alone. Instead, these cells may lack specific auxiliary factors required to propagate Type 2 MM sCJD strains in a less permissive replication environment, or the current assay may fail to detect these prions at lower concentrations; MM sCJD strains are significantly less protease-resistant than MV/VV strains and contain a higher proportion of PK-sensitive material (22). Future screening of HuPrP(M/MV129) subclones using orthogonal detection methods, such as alternative protease digestion (52) or seeding assays, may resolve this. Ultimately, a panel of distinct cell lines will likely be required to capture the full diversity of human prion strains.

The EKV cell line demonstrates that the propagation of bona fide human sCJD prion infectivity is not restricted to postmitotic neural environments, but can be sustained in dividing cells given the appropriate molecular compatibility. This faithful recapitulation of sCJD strain biology and infectivity in a scalable, experimentally tractable system provides a powerful platform to dissect the mechanisms of human prion uptake and replication, define the nature of human prion strains, and screen for therapeutics with direct relevance to human pathobiology.

## Materials and Methods

### Cell Lines and Routine Culture.

Unless otherwise specified, CAD5 cells and subclones thereof were cultured in Gibco Opti-MEM™ Reduced Serum Medium containing 10% Bovine Growth Serum (ThermoFisher Scientific, HyClone™) and 1% penicillin/streptomycin (ThermoFisher Scientific, 10,000 U/mL). Cells were routinely passaged 1:6 to 1:10 every 3 to 5 d via gentle trituration, and maintained in a humidified incubator (5% CO_2_, 37 °C).

### Retroviral Expression and Reconstitution of CAD5-KDB3 Cells With Human PrP.

Derivation of CAD5-KDB3 (KDB3) cells, in which the endogenous mouse PrP has been stably silenced by RNA interference, has been described in Bhamra et al. (19).

To reconstitute KDB3 cells with human PrP, a retroviral expression construct was prepared by inserting the human PrP open reading frame with valine at codon 129 into pLNCX2 (Clontech). The human PrP open reading frame was synthesized (Invitrogen GeneArt, ThermoFisher Scientific). In addition to valine at codon 129, the human signal peptide sequence (aa 1-22) was replaced with the mouse signal peptide, as we found this led to increased human PrP expression: mouse signal sequence MANLGYWLLALFVTMWTDVGLC (underlined amino acids indicate deviation between mouse and human amino acids). The human PrP coding region was sequence verified in two independent constructs (pLNCX2 mssHuPrPV129-8 and -24).

Both pLNCX2 constructs were packaged into recombinant retroviruses by transient transfection into Phoenix ecotropic cells (ATCC, LGC Standards, Middlesex, UK) in conjunction with vesicular stomatitis virus G (VSV-G) envelope expression vector (pMDG2) by FuGENE 6 Transfection reagent (Promega), according to the manufacturer’s instructions. We have found that pseudotyping the ecotropic retrovirus particles with the VSV-G protein increased the efficiency of stable transduction of CAD5 cells. Stable transduction of cells with pLNCX2 retroviruses was achieved by antibiotic selection using geneticin (G418, ThermoFisher Scientific, 400 µg/mL final concentration). 240 single cell clones were isolated for each construct and expanded. Clones were challenged with two sCJD-infected frontal cortex homogenates, and PrP^Sc^ detected in a modified Scrapie Cell Assay (SCA) format (described below). 58 initial clones were selected and reduced over serial assay to 4 candidate clones, based on the detection of PrP^Sc^ over at least one cell passage after dilution of signal from the residual inoculum. These clones were next challenged with two sCJD-infected frontal cortex homogenates [T3MV and T2MM strain types, London classification (6)] in the SCA and assayed in replicate wells to identify an optimal clone (81F9), which yielded PrP^Sc^ spots that were clearly identifiable and accumulated over serial passages (Fig. 1*A*). The optimal clone, 81F9, demonstrated reproducible PrP^Sc^ accumulation when challenged with a 3 × 10^−4^ dilution of T3MV sCJD frontal cortex (Fig. 1*B*).

### HPAs.

Frontal cortex 10% weight/volume homogenates were prepared by homogenization in sterile Dulbecco’s phosphate-buffered saline lacking Ca^2+^ and Mg^2+^ ions (D-PBS), using Duall tissue grinders. Initial human prion infectivity assays used the same format as the previously described Scrapie Cell Assay (11), but with modifications to enable detection of infectivity of human prions: 18,000 cells were seeded in a 96-well microplate and incubated for 24 h in 200 µL growth medium, after which 100 µL medium containing unfiltered prion-infected brain homogenate was added to each well. Cells were passaged twice weekly 1:6/1:8 at 3 to 4 d intervals, respectively. At confluence after passage 3, 4, and 5, 18,000 cells were filtered onto preactivated ELISPOT plates (MultiScreenHTS IP Filter Plate, 0.45 µm, Merck Millipore), fixed at 50 °C for 1 h, then lysed and incubated at 37 °C for 1 h in the presence of proteinase K (2.25 µg/mL), followed by 80 µL phenylmethylsulfonyl fluoride (PMSF, Sigma) for 10 min at room temperature. 80 µL of a 1:25,000 dilution of ≥250 units/µL stock benzonase solution (Merck Sigma) was then added and the plates incubated at 37 °C for 15 min. PrP^Sc^ was detected using 0.5 µg/mL mouse anti-PrP monoclonal antibody ICSM18 (D-Gen Ltd, UK), followed by alkaline phosphatase conjugated anti-mouse IgG1 and conjugate substrate (Bio-Rad). After drying plates, PrP^Sc^-positive spots were quantified using either the Zeiss KS ELISPOT system or the BioSys Bioreader®-7000-F imaging system.

For the optimized HPA for EKV cells, 2,500 to 5,000 EKV cells in 150 µL medium were added directly to wells of a 96-well microplate containing 150 µL brain homogenate diluted in medium. Cells were passaged as above, and 20,000 filtered per well for ELISPOT revelation after passages 4 and 5.

### Single Cell Cloning.

To derive a pool of single cell clones by limiting dilution, a CAD5-KDB3-derived parent cell line was resuscitated from cryopreservation and passaged twice in 2 µg/mL puromycin and 400 µg/mL G418. Cells were seeded in 10 cm dishes at a density of 150 cells/dish in 15 to 20 mL selection medium, and incubated for 10 to 12 d. Cultures were reviewed periodically, and single cell colony locations marked on the dish. After 10 to 12 d (approximately 5 to 7 doublings), growth medium was carefully changed and single cell colonies aspirated into individual wells of 96-well microplates. Wells were passaged twice at confluence, then expanded to triplicate plates; two replicate plates were cryopreserved and the third assessed for human prion susceptibility, as above. For each round of single cell cloning, between 400 to 1,000 clones were isolated, expanded, and screened for susceptibility to sCJD prions.

### Developing Cells Persistently Infected With Human Prions and Pilot Therapeutic Assays.

EKV cells were resuscitated, passaged twice in selection medium and challenged with 0.001% human prion-infected brain homogenate (either T3MV I4960, or T3VV 16058), followed by five passages to dilute out the inoculum. At confluence after passage 5, cells were seeded for single cell cloning as above, at a density of 1 cell per well in 300 µL medium, in two to four 96-well microplates. Cells were allowed to grow to confluence, after which the medium was replaced and cells passaged twice and expanded into triplicate plates. Two duplicate plates of each set were cryopreserved, and the final plate subjected to ELISPOT revelation to detect cells containing PrP^Sc^. Clones with the highest PrP^Sc^-positive spot number (infected EKV cells, iEKV) were expanded and cryopreserved in doubly sealed aliquots, in a dedicated vapor-phase liquid nitrogen storage tank exclusively for human prion-infected cells.

For pilot therapeutic assays, iEKV cells were resuscitated and passaged twice in growth medium. At confluence, 10,000 cells were seeded in 150 µL plain medium and allowed to adhere for 2 h. A dilution series of anti-PrP ICSM18 or BRIC222 (an IgG1 isotype control mouse monoclonal antibody) were added in 150 µL medium to appropriate wells. After 5 d of incubation, media was gently replaced and 7,000 cells per well filtered for ELISPOT or toxicity assay. Untreated iEKV cells, noninfected EKV cells, media-only wells, and DMSO-treated iEKV cells were used as controls. BRIC222 (IBGRL, Bristol, UK) was a kind gift from Dr. Azadeh Khalili-Shirazi. XTT toxicity assay (CyQuant XTT Cell Viability Assay, ThermoFisher Scientific) was undertaken as per the manufacturer’s instructions.

### Transmission Studies and Immunoblotting.

For animal inoculation with cell homogenates, 81F9 and KDB3 cells were challenged with 0.03% sCJD T3MV brain homogenate (I4960), or growth media only, and passaged six times (24 d postchallenge). At confluence, cells were pooled, resuspended in D-PBS at a concentration of 10 million cells per mL, ribolyzed, and frozen at −80 °C, for animal inoculation.

Tg152c mice were bred and maintained as described previously (53). Strict biosafety protocols were followed throughout. Inocula were prepared using disposable instruments for each inoculum, in a microbiological containment level 3 laboratory, and inoculations performed within a class 1 microbiological safety cabinet. Inocula were prepared in D-PBS, using Duall tissue grinders for human brain tissue, or ribolyzed in a Precellys®24 tissue homogenizer. Mice were culled when clinical signs of scrapie sickness were detected, or at 600 d postinoculation, as described previously (53).

Pooled lysates from sCJD-challenged and unchallenged 81F9 cells, and challenged KDB3 (PrP-knockdown) cells were inoculated into groups of Tg(HuPrP129V^+/+^*Prnp^0/0^*)-152 congenic mice. Tg152c mice express human PrP homozygous for valine at codon 129 at 4 to 8 times the level of normal human brain, on a mouse *Prnp*-null background (24), and are susceptible to all human prion strains (20). As a positive control, a separate group of Tg152c mice were inoculated with 1% I4960 sCJD T3MV frontal cortex homogenate. Mice were monitored for clinical signs of prion disease and culled when these were identified, or at experimental endpoint (600 d postinoculation, DPI).

For neuropathology and immunohistochemical analysis, transgenic mouse brains fixed in 10% buffered formal saline were immersed in 98% formic acid for 1 h. Following further washing in 10% buffered formal saline, tissue samples were processed and embedded in paraffin wax. Serial sections of 4 μm nominal thickness were prepared. Deparaffinized sections were analyzed for abnormal human PrP deposition on a Ventana Discovery XT automated IHC staining machine (Roche Tissue Diagnostics). In brief, sections were treated with cell conditioning solution (Discovery CC1; Roche Tissue Diagnostics) at 95 °C for 30 min, followed by treatment with a low concentration of protease (Protease 3; Roche Tissue Diagnostics) for 16 min. Anti-PrP monoclonal antibody 3F4 (BioLegend) was used in conjunction with a biotinylated polyclonal rabbit anti-mouse immunoglobulin secondary antibody and Ventana proprietary detection reagents utilizing 3,3′-diaminobenzidine tetrahydrochloride as the chromogen (DAB Map Detection Kit). Conventional methods on a Gemini AS Automated Slide Stainer (Thermo Fisher Scientific) were used for hematoxylin and eosin (H&E) staining. Positive controls for staining were used throughout. All slides were digitally scanned on a Hamamatsu NanoZoomer 360 instrument, and images captured from the Hamamatsu NZconnect image management software for the preparation of light microscopy images.

Samples were processed for immunoblot detection of proteinase K-resistant PrP as described previously (54). Human tissue samples were incubated for 1 h at 37 °C in 50 µg/mL proteinase K, and transgenic mice or cell lysate samples in 100 µg/mL proteinase K. CAD5 cell lysates were prepared from frozen pellets (10 million cells), resuspended in 1 mL D-PBS, ribolyzed, and immediately processed for immunoblotting.

### Statistical Analysis and Ethics.

All statistical analyses were performed using Graphpad Prism 9. All data referenced in this work are available on reasonable request. Ethical approval was granted by the local ethics board, and NHS Research Ethics Committee for human sample collection. All human tissue samples were deidentified prior to use.

## Data Availability

All study data are included in the main text.
